# Urinary neopterin levels increase and predict survival during a respiratory outbreak in wild chimpanzees (Taï National Park, Côte d’Ivoire)

**DOI:** 10.1038/s41598-018-31563-7

**Published:** 2018-09-06

**Authors:** Doris F. Wu, Verena Behringer, Roman M. Wittig, Fabian H. Leendertz, Tobias Deschner

**Affiliations:** 1 Department of Primatology, Max Planck Institute for Evolutionary Anthropology, Deutscher Platz 6, 04103 Leipzig, Germany; 2 Project Group Epidemiology of Highly Pathogenic Microorganisms, Robert Koch-Institut, Seestraße 10, 13353 Berlin, Germany; 3 Taï Chimpanzee Project, Centre Suisse de Recherches Scientifiques, BP 1303 Abidjan 01, Côte d’Ivoire

## Abstract

Monitoring immune system activation of wild animals has garnered increasing interest within the field of ecological immunology, leading to an urgent need for non-invasive biomarkers measuring these changes. Urinary neopterin, a marker of the cell-mediated immune response, is validated as an immune-related biomarker in captive and laboratory animals. However, wild animals naturally host higher and chronic pathogen loads. Therefore, detection and quantification of additional infections via neopterin might not be possible against the background of a chronically challenged immune system. To assess the suitability of urinary neopterin in wild animals, we measured neopterin corrected for specific gravity with an enzyme immunoassay in 185 samples collected before, during and after a respiratory disease outbreak in 28 individuals from a group of wild chimpanzees (Taï National Park, Côte d’Ivoire). Urinary neopterin levels were significantly higher during periods when individuals showed respiratory symptoms versus before and after the outbreak. Furthermore, urinary neopterin levels were significantly higher in individuals that died, with higher levels already apparent before the outbreak, suggesting individuals may have an already activated immune system. Measuring urinary neopterin levels, with other biomarkers of energetic condition, stress challenges, and reproduction will contribute towards a deeper understanding of life-history trade-offs in wild animals.

## Introduction

Being able to measure variation in immune system activation of wild animals has both practical applications towards monitoring the health status of wild populations threatened by disease^1^, as well as providing theoretical groundwork within the field of ecological immunology^2^. However, animal physiologists first require the validation of biological markers that can accurately monitor changes in immune system activation^3^. Specifically, the identification of immune-related biomarkers within non-invasively collected samples is of utmost importance to monitor patterns of immune system activation in free-ranging animals. While non-invasive methods have been used extensively in measuring various endocrine markers within the context of behavioural ecology^4–6^, there has been a limited availability of such methods for monitoring immunity and its role in wildlife disease ecology^3^.

Traditional methods of health monitoring typically require invasive methods (e.g., blood draws), leading to undue stress while not being applicable or feasible for longitudinal studies of wild large-bodied animals^4,7^. Non-invasive means of health monitoring, such as using urinary dipsticks^8–10^, behavioural observations^11,12^, and visual inspection for ecto- and endo-parasites^13,14^, can lack the required level of precision, and therefore need to be performed in conjunction with other methods to provide meaningful insights into an individual’s health status.

Neopterin, a pteridine, is an early inflammation marker in the cell-mediated immune response (Th1-type)^15,16^. It is produced by monocytes/macrophages, with production stimulated by interferon-gamma^17^. In humans, increases in serum neopterin levels have been described in reaction to several viral and bacterial infections, with elevated levels also observed in chronic infections^18^. Changes in neopterin levels are strongly associated with disease progression, severity and outcome^16,19,20^— with higher levels typically corresponding to a higher probability of mortality^18,21^. As neopterin is released as part of the activation of the non-specific immune response, it can also serve as a useful marker in monitoring immune system activation in the context of subclinical, unknown pathogens or diseases^18^.

Given its reliable application in the study of immune system activation in humans, neopterin has shown promise and garnered the interest of animal physiologists^3,22^, with particular note that neopterin is chemically stable, even under field conditions^7,22^, and released with no significant diurnal variation^18,23^. It is also detectable in several matrices including serum, saliva, and urine—with urinary levels correlating with serum measurements^3,15,16^, allowing for non-invasive collection. Therefore, measuring urinary neopterin levels has the potential to be of major diagnostic value in measuring and monitoring immune system activation in response to several known and unknown pathogens, as well as systemic conditions, in wild animals.

In zoo, laboratory, and semi-wild non-human primates, urinary neopterin has been successfully validated as a measure for immune system activation^22–24^. For example, in zoo bonobos (*Pan paniscus*), urinary neopterin levels exhibited a significant increase in response to observed respiratory infection symptoms^23^. Furthermore, in an experimental study with SIV-infected rhesus macaques (*Macaca mulatta*), elevated urinary neopterin levels were observed corresponding to persistent acute infections^21^. While showing clear responses to disease, the potential role of urinary neopterin in the study of life-history traits, however, has just recently been explored. Urinary neopterin levels of semi-free ranging Barbary macaques (*Macaca sylvanus*), were observed to increase with subject’s age, showing the potential of urinary neopterin measurements in research on immunosenescence in wildlife^24^.

For wild animal populations, it is suggested that the immune system is permanently challenged compared to captive animals as individuals often harbour multiple infections and are under constant pathogen pressure^25^. Such challenged immune systems are therefore expected to respond differently to novel and/or acute infections^26,27^. For example, humans and animals in tropical and rural environments that had elevated levels of chronic parasitic infection exhibited a decrease in vaccine efficacy; this was attributed to the chronic infections leading to an altered immune response^28^. Additionally, observation of various bird species in wild and captive settings exhibited different immune strategies based on their environments, which may reflect variability in energetic condition and pathogen load^26,27,29^. Therefore, patterns of immune system activation observed in captive settings might differ from those observed in response to an infection in wild individuals^26,27,29^. It is therefore possible that detection and quantification of an additional infection via certain immune-related biomarkers might be impossible against the background levels of an already chronically challenged immune system. A comparison of urinary neopterin levels across several studies in humans^17,30^ indicates that individuals living in environments with lower levels of pathogen prevalence do exhibit lower urinary neopterin levels. Therefore, it is necessary to determine whether an immune-related biomarker validated in captive animals, such as urinary neopterin, can also indicate changes in the immune system of wild animals.

Immune system activation is also a dynamic process with time delays observed between infection and the response of different components of the immune system^31^. Previous experimental studies measuring neopterin in humans and captive animals using frequent sampling show a temporal pattern with a delay in elevation following exposure, then a sharp, short-term peak measured at the end of the incubation period and before the production of specific antibodies, followed by a slow decline to baseline^3,18,32^. As the timing and magnitude of the peak varies between individuals and type of infections^3,18,21^, detecting changes in immune system activation during an acute outbreak in wild populations that often have limited sample availability adds an additional challenge. In this study, we aim to determine whether urinary neopterin levels are a valid non-invasive biomarker of immune system activation through monitoring changes in neopterin levels around a severe respiratory outbreak in a group of habituated wild chimpanzees of the Taï Chimpanzee Project in the Taï National Park, Côte d’Ivoire. We also aim to determine whether urinary neopterin levels correspond to observed signs of illness and can predict likelihood of survival.

## Material and Methods

### Study site and subjects

Data were collected between February 2009 and September 2010 in Taï National Park, Côte d’Ivoire. The study site is in an evergreen rainforest, which experiences an average annual rainfall of 1800 mm and average temperatures between 24 and 28 °C^33,34^. The site has two rainy (March-June, September-October) and two dry seasons (July-August, November-February)^33^.

The habituated South community of chimpanzees is part of the Taï Chimpanzee Project (established in 1979) and has been regularly followed since the 1980s^35^. At the beginning of the presented study, the group comprised of 37 individuals with 18 males (6 infants, 3 juveniles, 3 adolescents, 6 adults) and 19 females (7 infants, 2 adolescents, 10 adults) (age-sex class see: [35]).

### Respiratory Outbreak

In November 2009, a respiratory outbreak of high morbidity was observed in the group (Table 1). Signs of illness (e.g., coughing, lethargy, and nasal discharge)^36^ were first observed in five individuals on November 26^th^, and progressively spread throughout the group, peaking on November 30^th^ when 73% (n = 27) of the observed individuals exhibited disease symptoms. The first day when all individuals were observed free of disease symptoms was on December 20^th^. Across the entire outbreak, 84% of individuals developed severe respiratory symptoms (85% of infants, 100% of juveniles and adolescents, and 75% of adults) (Table 1). Fourteen individuals died from the disease including eight adults (3 males, 5 females) and six infants (2 males, 4 females). Twelve individuals (7 adults, 5 adolescent and juveniles) with particularly severe disease symptoms were treated with a long-acting antibiotic shot (Extencilline, Sanofi-Aventis, France) through remote injection with nine surviving^36^ (Table 1).

**Table 1:** Summary of the morbidity/mortality during the respiratory outbreak (N = 37 individuals).

Sex	Age-class (number)	Sick	Died	Treated
M	Infant (6)	67%	33%	0%
	Juveniles (3)	100%	0%	33%
	Adolescents (3)	100%	0%	100%
	Adults (6)	50%	50%	33%
F	Infant (7)	100%	57%	0%
	Juveniles (0)	0%	0%	0%
	Adolescents (2)	100%	0%	50%
	Adults (10)	90%	50%	50%
Total		84%	38%	32%

Percentage of group members that were sick, died, and treated with antibiotics for each age-sex class is given.

Molecular analyses from necropsy samples of some deceased individuals allowed identification of the human respiratory syncytial virus (HRSV-A) as the major causative agent of the outbreak^36^. Lung samples obtained from two chimpanzees deceased during the outbreak also showed evidence of co-infection with the human respiratory bacterium *Streptococcus pneumoniae*, which in combination with a respiratory virus, can lead to higher rates of fatality^36^.

### Sample collection

To investigate whether urinary neopterin levels increased during the respiratory outbreak, urine samples (N = 185) were selected from the biobank collection, taken as part of the long-term health monitoring program^1^. Samples were selected for each individual based on behavioural observations and veterinary health reports of respiratory disease symptoms and divided into three sampling periods categorized as: (1) pre-outbreak (PRE): from February 2009 until an individual exhibited disease symptoms; (2) outbreak (OB): from the first day an individual showed disease symptoms until the day it was symptom-free; (3) and post-outbreak (POST): from convalescence until September 2010. If available, at least three PRE and POST control samples were included from February 2009 to September 2010 to determine an individual’s baseline asymptomatic (and assumed healthy) neopterin levels. However, not all individuals had equal or available sampling from all three sampling periods either due to early deaths during the outbreak or because sampling of juveniles and infants was not conducted in a systematic way (Table 2). Urine samples were available from 28 individuals; 18 of which had available samples when symptomatic. Males (N = 13) between 2 and 45 years old and females (N = 15) between <1 to 44 years old were tested.

**Table 2:** Summary of number of individuals (N = 28) and urine samples (N = 185) that contributed to the three study periods.

	PRE	OB	POST
# of individuals	28	18	18
# of samples	76	55	54
median ± interquartile range (Range) samples/individual	3 ± 1 (1–4)	1 ± 2 (0–10)	2 ± 3 (0–7)

For each period, the median ± interquartile range and range (min to max) of samples per individual are given.

### Laboratory methods

Urinary neopterin levels were determined using a commercial competitive neopterin ELISA (Neopterin ELISA, Ref. RE59321, IBL International GmBH, Hamburg, Germany), which had been previously validated for quantifying urinary neopterin in captive chimpanzees^23^. Urine samples were thawed, vortexed, centrifuged and diluted to 1:400 with the kit provided assay buffer. When measured concentrations were off the linear range of the assay, samples were re-diluted up to 1:1600 until they fell within this range. The ELISA assays were performed following kit instructions and as described in [23]. All samples, standards, and controls were measured in duplicates with results expressed in nmol/L. The inter-assay variation of nine plates was 9.1% for high- and 9.9% for low-quality controls. Intra-assay variation was 6.1% for high- and 8.6% for low-quality controls.

Urinary specific gravity (SG) was measured using a digital handheld refractometer (TEC, Ober-Ramstadt, Germany). The population average for all chimpanzees measured was 1.017. To correct for variation in volume and concentration, final urinary neopterin levels were expressed in urinary neopterin (nmol/L) corrected for SG (corr. SG)^37^.

### Statistical analyses

To explore factors explaining variation in urinary neopterin levels (nmol/L corr. SG), a Linear Mixed Model (LMM)^38^ was fitted in R v3.3.1^39^ using the R package lme4 v1.1–17 (function lmer)^40^. The total sample size for these analyses was 185 samples from 28 individuals. The response variable, urinary neopterin levels (nmol/L corr. SG), was log-transformed. The full model included as fixed effects sampling period (PRE, OB, POST), sex, age, collection time, whether the individuals were treated with antibiotics, and whether they survived. Age and collection time were z-transformed to a mean of zero and standard deviation of one. Individual was included as a random intercept effect with random slopes for age and collection time within individual^41^. To determine the effect of sampling period, the full model was compared to the null model^42^ lacking this fixed effect, but being otherwise identical, using likelihood ratio tests (R-function anova with argument “test” set as “Chisq”)^42^. We tested the significance of individual predictors using likelihood ratio tests comparing the full model with models lacking them one at a time^41^. We assessed model stability by excluding individuals one at a time and comparing model estimates derived from these data with those of the full dataset. This test revealed the model to be stable. To test for collinearity, Variance Inflation Factors (VIF)^43^ were determined using the R-package car v.3.0-0 (R-function vif)^44^ applied to a linear model lacking the random effects. This revealed that collinearity (VIF scores <1.8) was not an issue^43,45^. Pairwise comparisons between sampling period on urinary neopterin levels were conducted using the R-package multcomp v1.4–8 (R-function glht)^46^ that provides p-values based on Wald approximation.

In a second model, we tested whether changes in urinary neopterin levels differed between individuals who survived or died during the outbreak period, using a reduced dataset because urine samples collected during the POST period and samples collected after antibiotic treatment needed to be excluded^32,47,48^. Analysis was also restricted to only include samples before the 9^th^ day after showing symptoms, which corresponds to the last day when an animal died after showing symptoms (N = 5). This was to account for the fact that animals who survived exhibited decreasing neopterin levels towards the end of the outbreak and during convalescence, compared to individuals who died earlier during the outbreak when neopterin levels were still high (see Supplementary Fig. S1). The total sample size for this analysis was 86 samples from 26 individuals. In this model, sampling period (PRE and OB) was tested in an interaction with survival as an additional fixed effect. This interaction was added as individuals who did not survive may already have had compromised health before showing symptoms compared to those that survived. Animals that survived may also be able to mount a more appropriate immune response to infection versus animals that did not survive. The LMM included the same fixed effects as the previous model. To determine the effect of survival, the full model was compared to the null model^42^ lacking survival and its interaction with sampling period, but being otherwise identical, using likelihood ratio tests (R-function anova with argument “test” set as “Chisq”)^42^. Neither model stability nor collinearity (VIF scores <1.4 for non-interaction terms)^43,45^ were an issue.

### Ethical Statement

This study only made use of non-invasive samples. Data and sample collection and transport was approved by the Ministry of Environment and Forests, Ministry of Research, and Office Ivoirien des Parcs et Réserves in Côte d’Ivoire. All experiments were performed in accordance with relevant guidelines and regulations.

## Results

**Figure 1:**
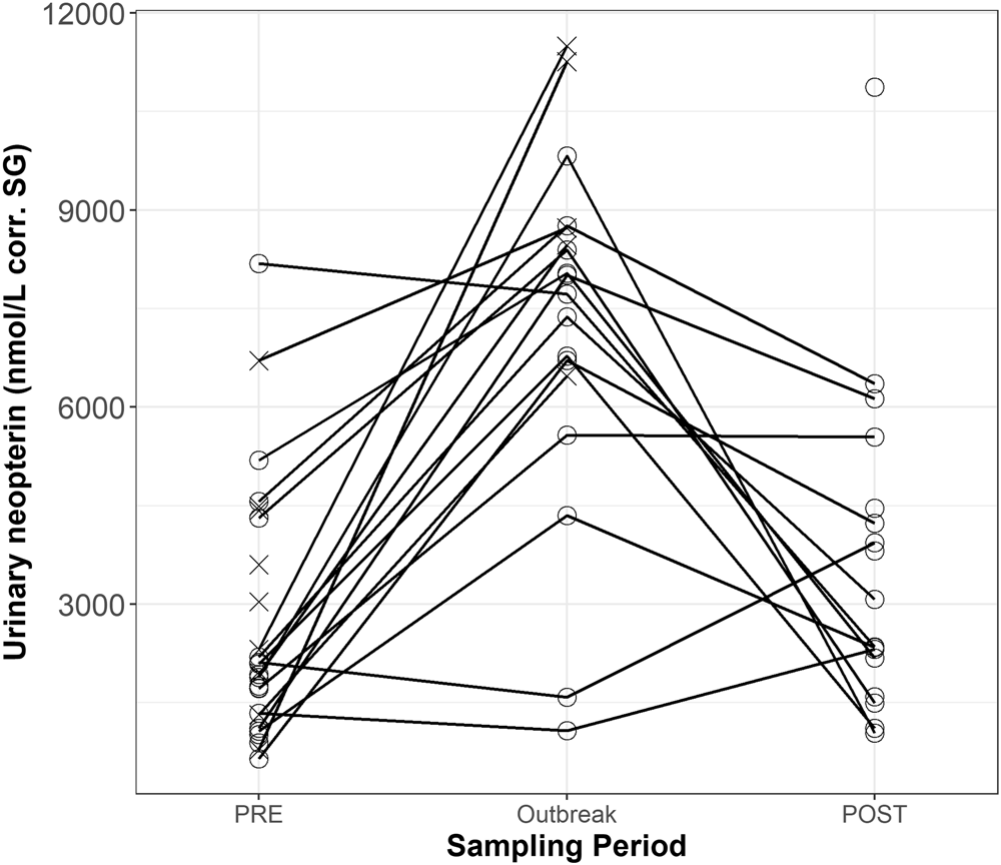
Average individual urinary neopterin (nmol/L corr. SG) levels during the study sampling periods (N_PRE_ = 28, N_OB_ = 18, N_POST_ = 18 individuals) for 185 samples. Cross marks represent individuals who did not survive (N = 10); open circles represent individuals who survived (N = 18). No line is shown for individuals with non-continuous sampling (N = 5).

Sampling period significantly predicted urinary neopterin levels (full-null model comparison, likelihood ratio tests: χ2 = 45.450, df = 2, P < 0.001). Urinary neopterin levels in samples collected when individuals were symptomatic during the outbreak (OB) (mean ± SD = 7952.5 ± 3797.9 nmol/L corr. SG) were significantly higher than in samples collected PRE (mean ± SD = 3220.0 ± 3962.9 nmol/L corr. SG) and POST (mean ± SD = 3453.2 ± 2830.8 nmol/L corr. SG) outbreak (Table 3, Fig. 1). The post-hoc comparison showed no significant difference in urinary neopterin levels between PRE and POST outbreak urinary neopterin levels (Table 4). During the OB, urinary neopterin levels (N = 18 individuals) increased from PRE levels by an average of 3.3, and up to 10.4 in one individual who survived (Fig. 1).

**Table 3:** Results of the LMM model (N = 185 samples) testing the impact of sampling period on urinary neopterin levels (nmol/L corr. SG) in wild chimpanzees (N = 28).

	Estimate	SE	*χ* ^2^	df	P
Intercept	9.252	0.232			
POST to OB	−1.154	0.194	45.450	2	<0.001**
PRE to OB	−1.367	0.188			
Survived	−0.463	0.195	5.402	1	0.020*
Sex	0.151	0.153	0.964	1	0.326
Age^(1)^	−0.072	0.078	0.806	1	0.369
Collection time^(1)^	−0.141	0.066	3.614	1	0.057
Antibiotic Shot	−0.436	0.249	2.495	1	0.114

^1^z-transformed to a mean of zero and standard deviation of one; mean (SD) of original variables were 17.61 (12.47) years and 11.08 (3.58) hours, respectively.

The full model included sampling period (PRE, OB, POST), whether individuals survived, sex, age, collection time, and whether individuals were treated with an antibiotic shot as fixed effects. Individual was included as a random intercept effect with random slopes for age and collection time.

**Table 4:** Post-hoc results of the LMM analyses (N = 185 samples) comparing the three sampling periods.

	Estimate	SE	*z-*value	P
PRE to OB	−1.367	0.188	−7.258	<0.001**
PRE to POST	−0.214	0.168	−1.268	0.207
OB to POST	1.154	0.194	5.962	<0.001**

A pairwise comparison between sampling periods on urinary neopterin levels (nmol/L corr. SG).

**Figure 2:**
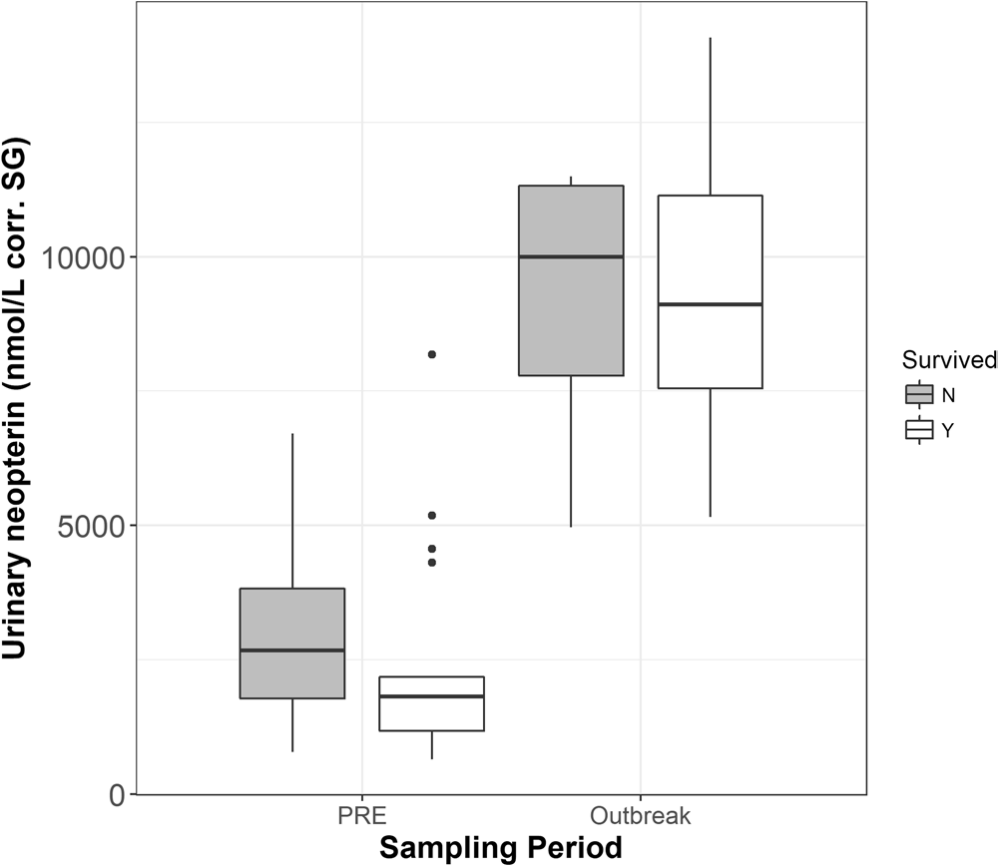
Average individual urinary neopterin (nmol/L corr. SG) levels before (N_PRE_ = 26) and during the outbreak (N_OB_ = 11) for chimpanzees who died (N = 8) and those who survived (N = 18) during the outbreak (N = 86 samples). Grey boxes represent those who died during the outbreak; white boxes represent those who survived. The median is indicated by the thick horizontal black line with the interquartile range represented within the boxes. The vertical lines indicate the upper and lower whisker with points as outliers.

The reduced model showed that animals who died had significantly higher urinary neopterin levels than animals who survived (full-null model comparison, likelihood ratio tests: χ2 = 7.747, df = 2, P < 0.021) (Table 5, Fig. 2). However, there was no significant effect of the interaction between health period and survival (χ2 = 0.891, df = 1, P < 0.345) on urinary neopterin levels. Compared to individuals who survived, individuals who died had higher urinary neopterin levels both before the start of the outbreak (mean ± SD = 4308.5 ± 5414.9 nmol/L corr. SG vs mean ± SD = 2384.7 ± 2014.4 nmol/L corr. SG), and during the beginning of the outbreak (mean ± SD = 9541.9 ± 3351.0 nmol/L corr. SG vs mean ± SD = 9101.2 ± 3004.4 nmol/L corr. SG) (Fig. 2).

**Table 5:** Results of the reduced LMM model (N = 86 samples) on the impact of survival on urinary neopterin levels (nmol/L corr.SG) in wild chimpanzees (N = 26).

	Estimate	SE	*χ* ^2^	df	P
Intercept	9.168	0.210			
Sampling Period	−1.355	0.191	38.226	1	<0.001**
Survived	−0.509	0.172	6.856	1	0.009*
Sex	0.343	0.165	3.398	1	0.065*
Age^(1)^	−0.100	0.085	1.314	1	0.252
Collection time^(1)^	0.020	0.087	0.053	1	0.818

^1^z-transformed to a mean of zero and standard deviation of one; mean (SD) of original variables were 18.52 (13.51 years and 10.95 (3.86) hours, respectively.

The full model included sampling period (PRE, OB), whether individuals survived, sex, age, and collection time as fixed effects. Individual was included as a random intercept effect with random slopes for age and collection time.

## Discussion

In this study, we monitored changes in urinary neopterin levels in wild chimpanzees during a respiratory outbreak of human respiratory syncytial virus (HRSV-A), with levels exhibiting a significant increase corresponding to observed respiratory symptoms. The average increase in urinary neopterin levels observed in this group of wild chimpanzees was comparable to results seen in a within-animal comparison of zoo bonobos who developed respiratory symptoms^23^. No significant differences in urinary neopterin levels were found between samples collected before (PRE) and after (POST) the respiratory outbreak—indicating a return to an individual’s baseline neopterin levels after recovery. The results of this study, therefore, indicate that changes in urinary neopterin levels can be used as an indicator of the activation of the non-specific immune system response, not only in captive, but also in wild great apes.

We furthermore investigated whether animals that died during the outbreak already had elevated neopterin levels before the outbreak, which may be indicative of an already compromised immune system. The interaction between sampling period and survival was not significant, with average urinary neopterin levels being similar during the beginning of the outbreak between those that survived and died. This may be due to the low sample size of animals who died during the outbreak. Therefore, it is possible that the significantly higher neopterin levels of individuals who died was mainly driven by samples taken before the outbreak, indicating an already challenged immune system. Additionally, an analysis of necropsy samples from some individuals that died during the outbreak revealed a co-infection with *S. pneumoniae*, a bacterium that may lead to chronic asymptomatic infections that can prove fatal once an individual is weakened by an acute infection^36^. This suggests that, while the degree of increase between individuals who did and did not survive did not differ significantly, elevated urinary neopterin levels before and during the outbreak of those that did not survive may still be indicative of an unfavourable disease outcome as previously described in humans^18,49^. This was also seen in an experimental study inducing sepsis in baboons (*Papio* sp.)^50^, as well as an experimental study exposing rhesus macaques to SIV^21^.

Although the increase in neopterin levels during the outbreak was significant, there was a large overlap between PRE and OB levels, as well as a high degree of variation in the PRE levels across individuals (Fig. 1). This is in accordance with past studies on human urinary neopterin levels that found that the range of urinary neopterin in active tuberculosis infections overlapped with both those in latent infections, as well as controls without any history of disease^48^. The variations observed in this study may be the result of an already existing immune challenge faced before the outbreak, such as co-infections^36^. However, due to limited sample availability during the outbreak (i.e., only six individuals had ≥3 samples during the outbreak), the absolute peak in urinary neopterin may not have been detected within an individual, contributing to the large overlap between urinary neopterin levels of sick and healthy individuals. Therefore, further studies are needed to investigate the causes of variation in urinary neopterin levels across seemingly healthy individuals. Currently, on a practical level, and as shown in a study of captive apes^23^, it is not possible to define a range of values indicating “healthy” versus “unhealthy” neopterin levels. For the moment, a detection indicating a change in health with elevated levels seems to be only possible by performing a within-subject comparison^51–53^.

How energy is allocated varies throughout an individual’s lifetime, with life-history models predicting a trade-off between promoting growth, maintaining health, and maximizing reproduction^54–56^. In recent years, how the immune system may play a role in the evolution of life-history traits has attracted considerable interest^1,57,58^. Specifically, investigations into the effects of sociality on variation in immune system activation and, ultimately, lifetime reproductive fitness, has given rise to the field of ecological immunology^2^. Therefore, being able to accurately measure and monitor variation in an individual’s immune system activation throughout an animal’s lifetime in a natural environment can provide valuable insights on the trade-offs of immune activation in the context of ecoimmunology^23,59,60^. Increasing evidence suggests that individual health is a key factor in mediating links between sociality, fitness, and disease susceptibility^61^ with social factors also, in turn, influencing immune functions^62–64^. In particular, there has been evidence showing an altered immune response following certain stressors leading to a suppression of T-cell function^64^. For example, suppressed urinary neopterin levels were observed to correspond with periods of psychological stress in medical students^63^. In this context, the non-invasive measurement of neopterin within and between wild populations, in combination with biomarkers of energetic condition, stress and reproductive system activation, will lead to a deeper understanding of these life-history trade-offs^23,65^.

While this study validated urinary neopterin as a biomarker of immune system activation during a severe respiratory infection, this method may have additional practical implications for the field of wildlife and conservation biology. In particular, it may aid in monitoring non-lethal, chronic infections of low intensity such as malaria in great apes, which often lack obvious sickness behaviors^66^. Neopterin has also been shown in humans to increase after vaccination^32,47^. And, given the threat of infectious diseases in decimating wildlife populations^1,36,67^, it therefore has potential use in non-invasively detecting the effectiveness of vaccinations and treatments in wild animals.

In conclusion, the biomarker urinary neopterin allowed for the monitoring of immune system activation in a wild chimpanzee group during a severe respiratory outbreak. Until factors leading to high across individual variability in asymptomatic individuals have been identified, longitudinal sampling is required to determine an individual’s baseline levels and to account for potential variations that occur due to natural temporal fluctuations and seasonality. This is particularly important when monitoring wild individuals who most likely harbour chronic low-level infections or are asymptomatic carriers of various pathogens. Additionally, measuring urinary neopterin in wild populations, in combination with other biomarkers of health and condition, will increase our understanding on variations in life-history strategies and contribute towards the growing field of ecoimmunology.

## Electronic supplementary material

Supplementary Figure S1

## Data Availability

The R code and datasets analysed during the current study are available from the corresponding author on reasonable request.
