# Supplementary material for: Urinary neopterin levels increase and predict survival during a respiratory outbreak in wild chimpanzees (Taï National Park, Côte d’Ivoire)
Source: Sci Rep. 2018 Sep 6;8:13346. doi: 10.1038/s41598-018-31563-7 (PMC6127264; doi:10.1038/s41598-018-31563-7)
Supplement: Supplementary file 1 — Supplementary Figure S1 [file 41598_2018_31563_MOESM1_ESM.pdf]

Urinary neopterin levels increase and predict survival during a respiratory outbreak in wild chimpanzees (Taï National Park, Côte d'Ivoire)

Doris F. Wu*[1,2], Verena Behringer[1], Roman M. Wittig[1,3], Fabian H. Leendertz[2], and Tobias Deschner[1]

[1]Department of Primatology, Max Planck Institute for Evolutionary Anthropology, Deutscher Platz 6, 04103 Leipzig, Germany

[2]Project Group Epidemiology of Highly Pathogenic Microorganisms, Robert Koch-Institut, Seestraße 10, 13353 Berlin, Germany

[3]Taï Chimpanzee Project, Centre Suisse de Recherches Scientifiques, BP 1303, Abidjan 01, Côte d'Ivoire

*corresponding author: doris_wu@eva.mpg.de

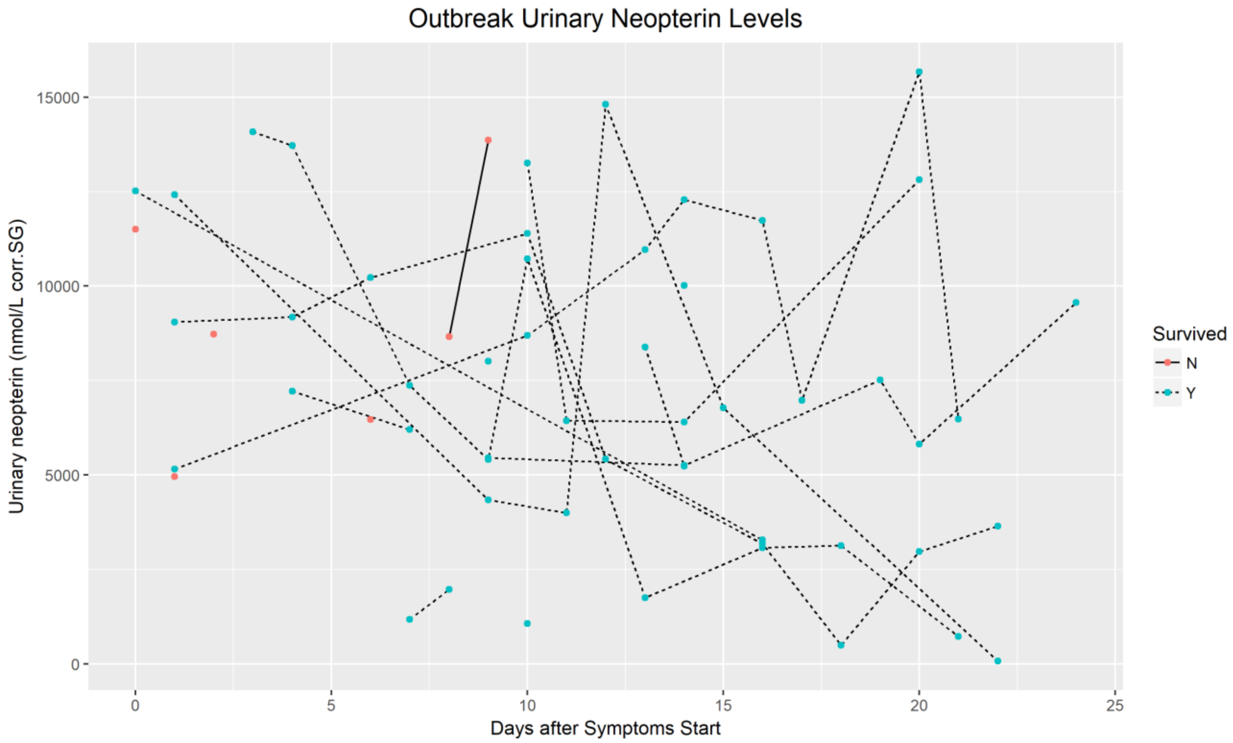

Figure S1. **Urinary neopterin levels (nmol/L corr. SG) across the outbreak period (N=58 samples).**

Samples were aligned for each individual to the first day of observed symptoms (day zero). Each line shows continuous sample points within an individual. Dashed lines and blue dots represent individuals who survived (N=13) and straight lines and red points indicate those that died (N=5).
